# Latitudinal variation and coevolutionary diversification of sexually dimorphic traits in the false blister beetle *Oedemera sexualis*

**DOI:** 10.1002/ece3.5101

**Published:** 2019-04-01

**Authors:** Daisuke Satomi, Chiharu Koshio, Haruki Tatsuta, Shin‐ichi Kudo, Yasuoki Takami

**Affiliations:** ^1^ Graduate School of Human Development and Environment Kobe University Kobe Japan; ^2^ Department of Biology Naruto University of Education Tokushima Japan; ^3^ Faculty of Agriculture University of the Ryukyus Okinawa Japan

**Keywords:** antagonistic coevolution, body size, latitudinal cline, sexual conflict, sexual selection

## Abstract

Sexual traits are subject to evolutionary forces that maximize reproductive benefits and minimize survival costs, both of which can depend on environmental conditions. Latitude explains substantial variation in environmental conditions. However, little is known about the relationship between sexual trait variation and latitude, although body size often correlates with latitude. We examined latitudinal variation in male and female sexual traits in 22 populations of the false blister beetle *Oedemera sexualis* in the Japanese Archipelago. Males possess massive hind legs that function as a female‐grasping apparatus, while females possess slender hind legs that are used to dislodge mounting males. Morphometric analyses revealed that male and female body size (elytron length), length and width of the hind femur and tibia, and allometric slopes of these four hind leg dimensions differed significantly among populations. Of these, three traits showed latitudinal variation, namely, male hind femur was stouter; female hind tibia was slenderer, and female body was smaller at lower latitudes than at higher latitudes. Hind leg sizes and shapes, as measured by principal component analysis of these four hind leg dimensions in each sex, covaried significantly between sexes, suggesting coevolutionary diversification in sexual traits. Covariation between sexes was weaker when variation in these traits with latitude was removed. These results suggest that coevolutionary diversification between male and female sexual traits is mediated by environmental conditions that vary with latitude.

## 1 INTRODUCTION

Sexually selected traits can diverge rapidly in response to a balance between natural and sexual selection, that is, a balance between the survival costs associated with bearing the trait and the benefits of reproductive success (Svensson & Gosden, 2007). This process may be influenced by environmental factors that can vary spatially (Svensson, Kristoffersen, Oskarsson, & Bensch, 2004). Variation in the degrees of natural and sexual selection between populations may also mediate relative investment in a sexually selected trait (Cayetano, Maklakov, Brooks, & Bonduriansky, 2011; Endler & Houde, 1995; Wilkinson, 1993). Directional sexual selection for an exaggerated sexual trait is expected to favor a larger body that enables disproportionate investment in the trait. However, because exaggerated sexual traits in males are typically costly to express and because both costs and benefits are likely to depend on environmental and individual (e.g., nutritional) conditions, sexually selected traits may exhibit high levels of condition dependence (Bonduriansky, 2007; Cothran & Jeyasingh, 2010; Cotton, Fowler, & Pomiankowaki, 2004). Thus, relative investment in an exaggerated sexual trait and the resultant diversification of size and allometry among populations are expected to more or less correlate with environmental variation.

Environmental variation may also influence the degree of reproductive competition between males and/or sexes via demographic processes (e.g., population density and sex‐related mortality), resulting in environment‐dependent sexual trait evolution. The intensity of sexual conflict and the evolutionary response to sexually antagonistic selection are expected to be greater in large and dense populations (Gavrilets, 2000). Thus, sexually antagonistic coevolution tends to escalate in populations at low latitudes with relatively mild environments (Arnqvist, Edvardsson, Friberg, & Nilsson, 2000), which favor increases in population size and density.

A major source of spatial environmental variation is latitude, which correlates with temperature, precipitation, and seasonality and has been shown to influence traits (Bergmann, 1848; Blanckenhorn & Demont, 2004; Blanckenhorn, Stillwell, Young, Fox, & Ashton, 2006). Variation in body and trait sizes among populations has also been attributed to factors that may covary with latitude, such as larval diet (Cassidy, Bath, Chenoweth, & Bonduriansky, 2014), host plant availability (Miller & Emlen, 2010), population density (Tomkins & Brown, 2004), and climatic stress (Mysterud, Meisingset, Langvatn, Yoccoz, & Stenseth, 2005). Latitudinal clines in body size were originally described for endotherms, such as birds and mammals, by Bergmann (1848), who proposed that, within a widespread taxonomic group, populations, species, and genera with larger body sizes are found in colder environments, usually located at higher latitudes, while the opposite is observed in warmer environments, usually situated at lower latitudes. This trend is also reported in a comparative study across exotherm animals, indicating that lower temperature leads to a larger body size because of the relationship between metabolic rate and temperature (Riemer, Anderson‐Teixeira, Smith, Harris, & Ernest, 2018). The opposite trend is also predicted in exotherms; because their growth depends on temperature, lower latitudes and altitudes (i.e., warmer environments) are associated with larger body sizes and higher latitudes and altitudes (i.e., cooler environments) are associated with smaller body sizes (Blanckenhorn & Demont, 2004; Blanckenhorn et al., 2006). Latitudinal clines in body size are found in various groups of animals (e.g., crustaceans, Timofeev, 2001; insects, Cushman, Lawton, & Manly, 1993; Sota, Takami, Kubota, & Ishikawa, 2000a; Sota, Takami, Kubota, Ujiie, & Ishikawa, 2000b; fish, Belk & Houston, 2002; Estlander et al., 2017; amphibians, Adams & Church, 2008; Ashton, 2002; and reptiles, Ashton & Feldman, 2003). However, latitudinal variation in sexual traits has rarely been explored (Painting, Buckley, & Holwell, 2014; Romiti et al., 2017).

**Figure 1:**
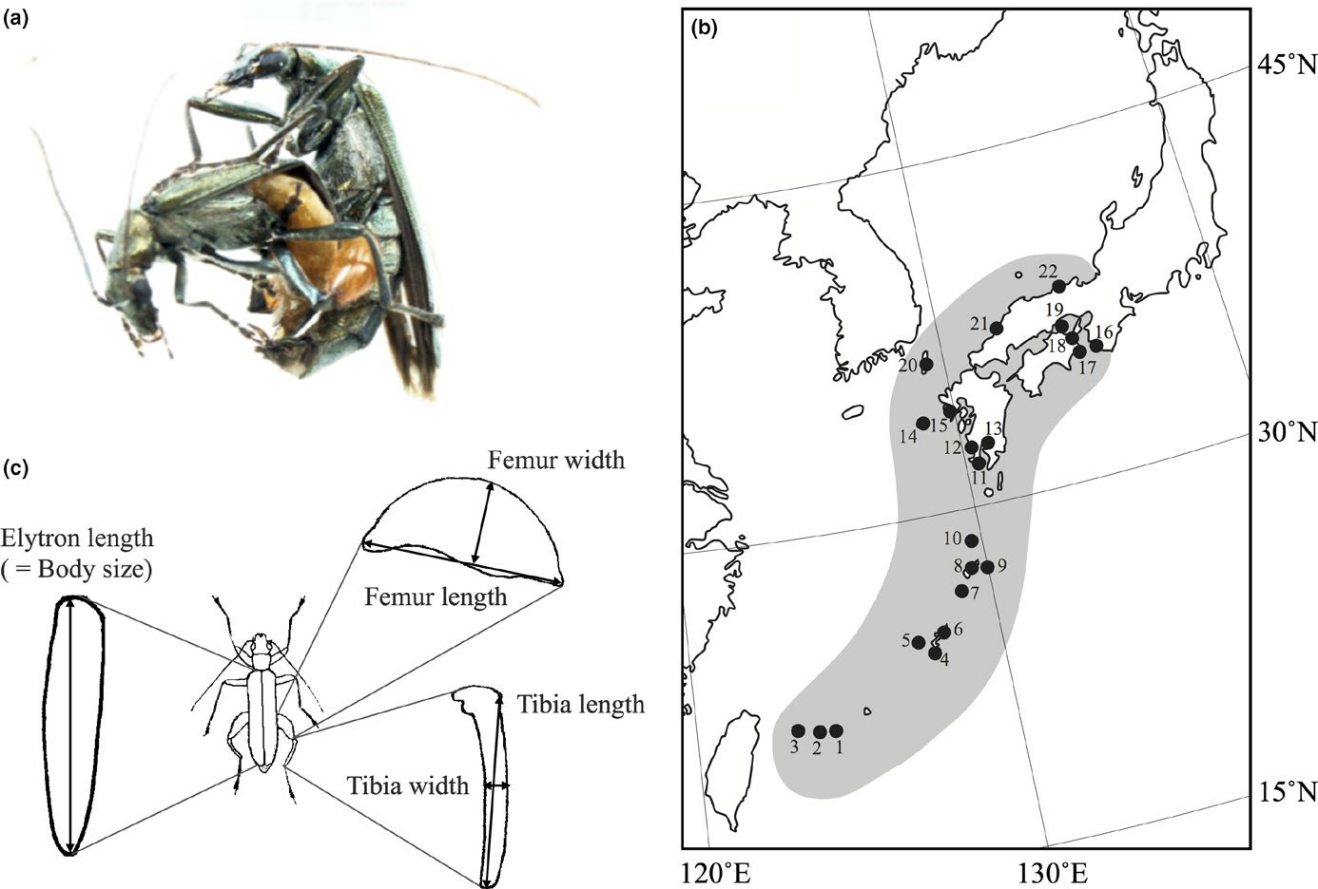
(a) A male *Oedemera sexualis* struggles to copulate with a female; the massive hind legs function as a female‐grasping apparatus. The female tries to kick‐off the mounting male using her hind legs. (b) Map showing the locations of the 22 sample populations of *O. sexualis*. Shading indicates the species distributional range. (c) Measurements of five morphological traits. EL: Elytron length (body size); FL: femur length; FW: femur width; TL: tibia length; TW: tibia width.

The purpose of this study was to examine whether and how the size, shape, and allometric slopes of sexual traits covary with latitude. To this end, we focused on the false blister beetle *Oedemera sexualis*, an ideal model system for evaluating geographic variation in exaggerated sexual traits. This species is widely distributed along latitudes, and its hind leg morphology is sexually dimorphic. The male has massive hind legs functioning as a female‐grasping apparatus, while female hind legs are slender and used to dislodge the mounting male by dashing and kicking him (Koshio et al., unpublished data; Figure 1a). Male–male combat using hind legs has never been observed. These facts suggest that the sexually dimorphic hind legs of this species play a role in the context of intersexual selection, or more probably sexual conflict. Here, we hypothesize that the environmental dependency of sexual and natural selection drives diversification in sexual traits among populations in different environmental regimes. This hypothesis predicts that sexual trait and its degree of variation (e.g., allometry) will vary along an latitudinal environmental cline. Additionally, if intersexual selection and/or sexual conflict are involved, it is expected that sexual traits will covary between the sexes across populations and that such a coevolutionary trajectory may also be related to latitude. We investigated latitudinal variation in body size, hind leg morphologies, and their scaling relationships in 22 populations of *O. sexualis*. Additionally, we investigated covariation in hind leg morphologies between males and females to examine coevolutionary diversification between sexes. Based on these results, we discuss the environmental dependence of the evolution of sexual traits.

## 2 MATERIALS AND METHODS

### 2.1 Organism, sampling, and measurements

*Oedemera sexualis* (Coleoptera: Oedemeridae) is endemic to the Japanese Archipelago and distributed from the southern end of the Ryukyu Islands to the western part of Honshu mainland (Svihla, 1999; Figure 1b). Adults aggregate on flowers (e.g., *Castanopsis sieboldii* and *Melia azedarach*) in coastal areas from the end of March to June, and the active season of a single population is restricted to about 1–2 months and varies along latitudes. In total, 1,643 individuals (male, *N* = 901; female, *N* = 742) were collected from 22 localities from 2002 to 2016 (Figure 1b; Supporting information Table S1). These localities were scattered across a latitudinal range of 11.17° (24.22°–35.39°), which covers most of the distributional range of *O. sexualis*.

To evaluate phenotypic variation in sexual traits within and between populations, male and female hind legs were measured, including the hind femur length (FL), hind femur width (FW), hind tibia length (TL), and hind tibia width (TW), using a digital sight imaging controller (Nikon DS‐L2) attached to a binocular microscope to the nearest 0.01 mm (Figure 1c). Elytron length (EL) was also measured as a proxy of body size. These traits were measured bilaterally, and two measurements of the left and right parts were averaged prior to subsequent analyses. These measurements were confirmed to be highly repeatable based on three‐time repeated measures of 20 individuals in the Yambaru population (site 6) (*R* ^2^ > 0.99 for all eight traits [four for each sex], ANOVA, *p* < 0.001). All measurements were log_10_‐transformed prior to subsequent analyses.

Three populations in the Amami Islands (sites 7, 8, and 9) constituted significant outliers with exceptionally narrow male hind FWs (Smirnov–Grubbs test, *p* < 0.03; Supporting information Figure S1). Male mating behavior in these populations differed conspicuously from that in other populations (Koshio et al., unpublished data), suggesting these populations evolved along a trajectory that was qualitatively different from that of other populations. Therefore, these three populations were excluded from the following analyses.

### 2.2 Climatic variation

Latitude is expected to influence various environmental parameters, such as the phenology of flowering plants, which are food resources and the arena of mating interaction for this species. Correlations between climate variables and latitudes were evaluated across the distributional range of this species. Climates of the study sites were evaluated by annual mean temperature, minimum temperature, maximum temperature, and annual mean rainfall, which were obtained from the Japan Meteorological Agency (data from 1981 to 2010, http://www.jma.go.jp/jma/index.html). As a result, annual mean, minimum, and maximum temperatures were significantly negatively correlated with latitude, indicating that latitude is a good proxy of climates in this study area (Supporting information Table S2).

### 2.3 Morphological variation and allometry

To examine geographical variation in hind leg dimensions (FL, FW, TL, and TW) and body size (EL), generalized linear models (GLMs) were constructed with a normal distribution and identity link function, in which one of the traits was used as the objective variable and population was treated as an explanatory variable. Then, two explanatory variables (EL and an interaction between population and EL) were added to the models of hind leg dimensions to examine geographical variation in the relative size of hind leg parts and their allometric slopes. A significant interaction between population and EL was expected if allometric slopes differed among populations.

For the analysis of latitudinal variation, allometric slopes of the hind leg dimensions were estimated in individual populations. Analytical techniques for allometric relationships have been debated; these debates are mostly focused on the choice between ordinary least‐squares (OLS) regression, which is simple but was originally used to predict Y from X or to suggest X causes a change in Y, and other types of regression, which determine a best fit line to approximate the relationship between two variables involving errors (Smith, 2009; Warton, Wright, Falster, & Westoby, 2006). In this study, both OLS and standard major axis (SMA) regressions were used to determine the allometric slopes of the hind leg sizes in each population using the package smatr version 3.2.3 in R (Warton, Duursma, Falster, & Taskinen, 2012). The deviation from zero was tested by consulting the 95% confidence intervals of the standard major axis (SMA) slopes. Since the estimated slopes were strongly correlated between OLS and SMA in all traits (*r*_19_ = 0.76–0.97, *p* < 0.001), only the SMA results are shown. SMA has been frequently used in recent studies on the latitudinal cline of sexual traits (Painting et al., 2014; Romiti et al., 2017) and is thus useful for making comparisons.

### 2.4 Latitudinal variation

Because the femur and tibia of the hind leg constitute a functional unit involved in mating, principal components were calculated to capture overall variation in hind leg morphology. Principal component analysis (PCA) was performed based on the variance–covariance matrix between population mean values of the four hind leg measurements (Supporting information Table S3; Figure S2). Population means were used to remove the effect of within‐population variation on PCA, because sample sizes varied among populations and determining variation among populations was the purpose of the study. PCA was performed separately for males and females to keep the PC scores independent between sexes. In males, the first principal component (PC1, 81.9%) showed positive loadings of all four traits; and the second principal component (PC2, 14.6%) showed a positive loading of FW and negative loadings of the other three traits (Table 1). In females, PC1 (89.7%) also showed positive loadings of all four traits; PC2 (7.7%) showed positive loadings of FL, FW, and TL and negative loading of TW (Table 1). Male and female PC1s were significantly correlated with male and female ELs, respectively, while PC2s were not (PC1 vs. EL, male: *r*_19_ = 0.85, *p* < 0.001, female: *r*_19_ = 0.90, *p* < 0.001; PC2 vs. EL, male: *r*_19_ = −0.44, *p* = 0.057, female: *r*_19_ = 0.02, *p* = 0.93). Therefore, the PC1s and PC2s may represent hind leg sizes and shapes, respectively. These PC scores were used to verify latitudinal variation and covariation between the sexes.

**Table 1:** Principal component analysis of four hind leg traits in *Oedemera sexualis*

	Male		Female	
	PC1	PC2	PC1	PC2
Eigenvalue	0.0023	0.0004	0.0024	0.0002
Variance (%)	81.92	14.55	89.66	7.68
Cum. variance (%)	81.92	96.50	89.66	97.34
FL	0.369	−0.261	0.393	0.538
FW	0.631	0.697	0.494	0.391
TL	0.330	−0.627	0.406	0.311
TW	0.597	−0.230	0.661	−0.727

Note

FL, hind femur length; FW, hind femur width; TL, hind tibia length; TW, hind tibia width.

To examine latitudinal variation in hind leg morphologies, body sizes and allometric slopes of hind leg dimensions, the effects of latitude and island/mainland on hind leg PC1 and PC2, EL, and the allometric slopes in each sex were analyzed by GLMs. Trait size may be influenced by an island effect owing to limited resources on small islands, invasion history, and subsequent genetic bottlenecks (Foster, 1964; Palmer, 2002).

### 2.5 Covariation between sexes

To examine coevolutionary diversification between male and female sexual traits, the association of hind leg PC1 and PC2 and body sizes (EL) between sexes was examined by Pearson's correlation coefficients. Additionally, the effect of latitude on coevolutionary diversification between sexes was checked by using correlations between the residuals of the traits from linear regressions on latitude.

## 3 RESULTS

### 3.1 Morphological variation and allometry

All the body and hind leg measurements differed significantly among populations (Table 2; Supporting information Figure S3). Allometric slopes of male FW and TL differed significantly among populations, as indicated by significant interactions between population and EL (Table 2), while allometric slopes of other male traits and those of all female traits did not significantly differ among populations (male: FL, *F*_21, 857_ = 1.43, *p* = 0.10; TW, *F*_21, 857_ = 1.20, *p* = 0.25; female: FL, *F*_21, 698_ = 1.45, *p* = 0.09; FW, *F*_21, 698_ = 1.22, *p* = 0.23; TL, *F*_21, 698_ = 1.24, *p* = 0.21; and TW, *F*_21, 698_ = 0.87, *p* = 0.63). Allometric slopes estimated in each population (Supporting information Table S4) were used in the following analysis.

**Table 2:** Generalized liner models explaining variation in male and female morphologies across 22 populations

Trait	Factors	Male			Female		
		*F*	*df*	*p*	*F*	*df*	*p*
EL (Body size)	Population	8.51	21,879	<0.001	3.46	21,720	<0.001
FL	Population	29.48	21,878	<0.001	18.80	21,719	<0.001
	Body size	7,077.13	1,878	<0.001	8,675.34	1,719	<0.001
FW	Population	405.42	21,857	<0.001	17.24	21,719	<0.001
	Body size	3,723.87	1,857	<0.001	2,545.54	1,719	<0.001
	Interaction	1.91	21,857	0.008			
TL	Population	8.05	21,857	<0.001	11.11	21,719	<0.001
	Body size	4,225.20	1,857	<0.001	6,913.56	1,719	<0.001
	Interaction	1.75	21,857	0.020			
TW	Population	40.64	21,878	<0.001	19.12	21,719	<0.001
	Body size	2,952.34	1,878	<0.001	2,154.72	1,719	<0.001

### 3.2 Latitudinal variation

**Figure 2:**
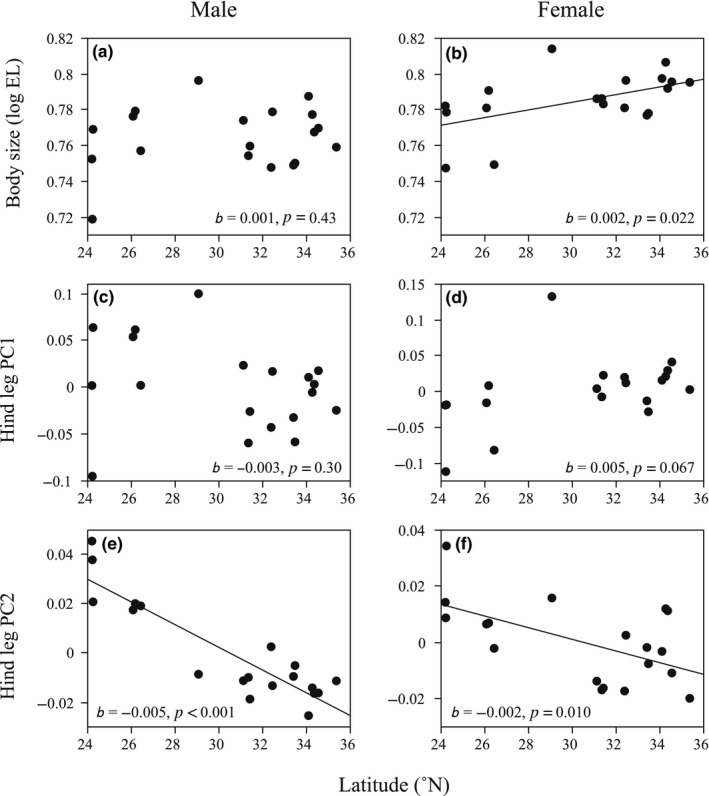
Latitudinal variation in (a) male and (b) female body sizes, and (c, e) male and (d, f) female hind leg shapes. Linear regressions show significant associations with latitude (*p < *0.05)

Male EL was not significantly associated with latitude, while female EL was significantly greater at higher latitudes (Table 3 and Figure 2a, b). Male and female hind leg PC1s were not significantly associated with latitude (Table 3 and Figure 2c, d), while male PC2 was significantly higher at lower latitudes (Table 3 and Figure 2e). The island effect was not significant in these GLMs (*p* > 0.151) and was excluded from the final models. Female hind leg PC2 was significantly higher at lower latitudes (Table 3 and Figure 2f), but this association was not significant after including the island effect (latitude, *F*_1,17_ = 2.26, *p* = 0.16; island effect, *F*_1,17_ = 5.35, *p* = 0.034). Allometric slopes of male FW and TL, which varied significantly among populations, were not significantly associated with latitude (Table 3).

**Table 3:** Tests for latitudinal variation in hind leg morphologies (PC scores), body size (EL), and allometric slopes of male hind legs (FW and TL) in *Oedemera sexualis*

Trait	Male				Female			
	*b*	*SE*	*F*_1,17_	*p*	*b*	*SE*	*F*_1,17_	*p*
EL (Body size)	0.001	0.001	0.65	0.43	0.002	0.001	6.35	**0.022**
Hind leg PC1	−0.003	0.002	1.13	0.30	0.005	0.003	3.84	0.067
Hind leg PC2	−0.005	0.001	72.15	**<0.001**	−0.002	0.001	8.37	**0.010**
Male FW slope	0.02	0.01	3.46	0.080				
Male TL slope	0.01	0.01	2.69	0.12				

Note

Significant results (*p* < 0.05) are shown in boldface.

### 3.3 Covariation between sexes

**Figure 3:**
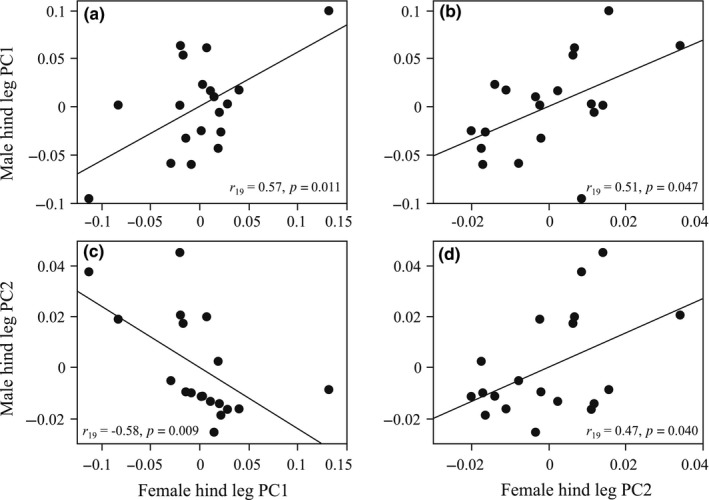
Covariation in hind leg morphology between sexes. (a) Male PC1 versus female PC1, (b) male PC1 versus female PC2, (c) male PC2 versus female PC1, (d) male PC2 versus female PC2. The fitted line shows significant correlations (*p < *0.05).

Hind leg morphologies significantly covaried between the sexes across populations (Figure 3). The male hind leg was large (as represented by male PC1) when the female hind leg was large (female PC1) and hind tibia was slender (female PC2); the male hind femur was wide (male PC2) when the female hind leg was small (female PC1) and hind tibia was slender (female PC2). When environmental effects were removed from PC2s (i.e., latitude in male PC2 and latitude and island/mainland in female PC2), these correlations were statistically marginal (female PC1 vs. residual male PC2, *r*_19_ = −0.45, *p* = 0.056) or not significant (male PC1 vs. residual female PC2, *r*_19_ = 0.38, *p* = 0.11; residual male PC2 vs. residual female PC2, *r*_19_ = −0.19, *p* = 0.44).

Hind leg morphology was significantly associated with the body size of the other sex (male PC1 vs. female EL, *r*_19_ = 0.50, *p* = 0.029; male PC2 vs. female EL, *r*_19_ = −0.61, *p* = 0.006; female PC1 vs. male EL, *r*_19_ = 0.73, *p* < 0.001), except for female PC2 (female PC2 vs. male EL, *r*_19_ = 0.23, *p* = 0.35). These associations showed that (a) the male hind leg was large and hind femur was slender when the female body was large and that (b) the female hind leg was large when the male body was large. When environmental effects on body size and sexual traits (i.e., latitude in female EL, and other traits were as shown above) were removed, the correlation between male PC1 and residual female EL was significant (*r*_19_ = 0.74, *p* < 0.001), but not that between residual male PC2 and residual female EL (*r*_19_ = −0.38, *p* = 0.11). The correlation between residual female PC2 and male EL remained not significant (*r*_19_ = 0.32, *p* = 0.19).

## 4 DISCUSSION

Although trait variation across geographic regions is a fundamental subject with a long history in evolutionary biology, the processes responsible for geographic variation in sexual traits are still only partially understood (Hasegawa & Arai, 2013; Kelly, Folinsbee, Adams, & Jennions, 2013; Monteiro & Lyons, 2012; Outomuro & Johansson, 2011; Painting et al., 2014; Romiti et al., 2017). In this study, we observed remarkable variation in body size, the dimensions of sexually dimorphic hind legs, and their allometric slopes across populations of *O. sexualis*. Male and female hind leg morphologies and female body size were significantly associated with latitude. The male hind leg was bigger and the hind femur relatively wider, while the female hind leg was bigger and the hind tibia relatively slenderer at lower latitudes than at higher latitudes. These results are concordant with our primary prediction. Additionally, an island effect was detected in the female hind leg shape (i.e., PC2), suggesting that factors other than latitudinal environmental variation may also be responsible for the variation in this female sexual trait.

The latitudinal covariation in hind leg morphologies between the sexes (Figure 3) provides insights into the process of sexual trait coevolution. *O. sexualis* males never fight together with their enlarged hind legs, unlike other species with robust male hind legs, such as coreid bugs (Eberhard, 1998; Mitchel, 1980; Miyatake, 1993,1997) and leaf beetles (Eberhard & Marin, 1996). Rather, the male hind legs are used to grasp a struggling female mate, and the female hind legs are used to kick‐off the mounting male (Figure 1a). The males with enlarged hind legs usually have a mating advantage (Koshio et al., unpublished data). Thus, it seems likely that sexual conflict contributes to the evolution of sexual traits in *O. sexualis*, and the covariation between the male and female hind leg morphologies is the result of antagonistic coevolution between sexes. Antagonistic coevolution between male grasping and female antigrasping structures is also found in *Gerris* water striders (Arnqvist & Rowe, 2002; Perry & Rowe, 2011).

The intensity of sexual conflict and evolutionary response to sexually antagonistic selection is expected to be greater in large and dense populations (Gavrilets, 2000). Thus, latitudinal variation in population sizes and densities would explain the latitudinal covariation between male and female sexual traits. Reduction or disappearance of covariation between the sexes after removing the effect of latitude and island suggests that this could be an apparent covariation in which male and female traits responded independently to environmental factors or colonization processes, or that coevolution between the sexes was strongly influenced by these factors. It may be difficult to distinguish true coevolution from apparent covariation. However, we also found some cases of sexual trait covariation independent of environmental factors, suggesting coevolution between the sexes may be a more plausible explanation. Quantitative evaluation of sexually antagonistic selections operating on the male and female traits is warranted to distinguish between these possibilities.

At higher latitudes, where cooler and drier climates presumably inflict a higher level of stress on individual development, a higher proportion of individuals would be expected to be affected by resource limitation, and there would be fewer overall resources to acquire and allocate to sexual traits. This may also explain the less developed hind legs in northern populations of *O. sexualis*. Sexually selected traits are sensitive to environmental variation and resource stress and therefore show a high level of condition dependence (Bonduriansky, 2007; Cothran & Jeyasingh, 2010). In stressful environments, secondary sexual trait size should be more variable within populations because genetic variation among individuals in the ability to acquire and allocate resources will become more pronounced as resources become limited (Cotton et al., 2004). Unlike population means of the traits, however, allometric slopes of male and female hind leg dimensions in *O. sexualis* were not clearly associated with latitude. In recent examples of latitudinal cline in exaggerated male traits used as weaponry in male–male combat, allometric slopes were associated with latitude (Painting et al., 2014; Romiti et al., 2017). Processes responsible for the differentiation of allometric slopes of sexual traits may differ between male–male competition and intersexual selection or sexual conflict.

We found that only females had larger mean body sizes at higher latitudes. Spatial variation in body size in a single sex can be attributed to sex‐specific responses to macro‐environmental gradients (e.g., the differential‐plasticity hypothesis; Fairbairn, 2005; Hu, Xie, Zhu, Wang, & Lei, 2010; Stillwell & Fox, 2007). Latitudinal variation in body size in only one sex frequently involves larger females toward the poles, and this may be explained by selection for fecundity (Cox, Skelly, & John‐Alder, 2003; Fitch, 1981; Litzgus & Smith, 2010). A fecundity advantage involves more offspring per reproductive bout during a short reproductive season (Cox et al., 2003). In *O. sexualis*, the period suitable for breeding may be shorter, and thus, oviposition frequency might be lower in northern populations. Consequently, the intensity of fecundity selection on females would be stronger in such populations.

To our knowledge, this is the first study demonstrating latitudinal covariation between male and female sexual traits. Our results provide novel insight into the environmental dependence of coevolution between male and female sexual traits, but there are still unsolved issues. We found three outlier populations in the Amami Islands (sites 7, 8, and 9) with exceptionally narrow male hind femurs (Supporting information Figure S1). These populations departed conspicuously from the trend in latitudinal variation, implying that other evolutionary processes producing distinctive morphologies were at work in this population. The relationships between hind leg morphology and mating behavior as well as the strength of sexual selection operating on hind leg morphology should be compared between these three and other populations. We detected an island effect on female hind leg morphology (PC2), suggesting that ecological (e.g., limited resources) and/or genetic (e.g., bottlenecks) effects were responsible for this difference in variation between islands and the mainland. Further ecological, behavioral and phylogeographic analyses will be required to improve our understanding of the evolution of geographical variation in sexual traits in *O. sexualis*.

## CONFLICT OF INTEREST

None declared.

## AUTHOR CONTRIBUTIONS

D.S., C.K., and S.K. conceived the study. D.S., C.K., H.T., S.K., and Y.T. collected the materials. D.S. performed the morphological measurement and analysis. D.S. drafted the manuscript and revised it in cooperation with C.K., H.T., S.K., and Y.T. All authors discussed the results and implications of the study at all stages.

## Data Availability

The DOI for our data is https://doi.org/10.5061/dryad.3fh816m
