# Supplementary material for: Latitudinal variation and coevolutionary diversification of sexually dimorphic traits in the false blister beetle Oedemera sexualis
Source: Ecol Evol. 2019 Apr 1;9(8):4949–57. doi: 10.1002/ece3.5101 (PMC6476772; doi:10.1002/ece3.5101)
Supplement: Supplementary file 4 [file ECE3-9-4949-s004.doc]

**Supplementary**

Table S1. Locality of populations and sample sizes.

| Number | Site | Type | Latitude (°N) | Longitude (°E) | Male | Female |
| --- | --- | --- | --- | --- | --- | --- |
| 1 | Ishigakijima | island | 24°22' | 124°08' | 40 | 20 |
| 2 | Iriomotejima | island | 24°24' | 123°48' | 20 | 12 |
| 3 | Yonagunijima | island | 24°27' | 122°58' | 88 | 80 |
| 4 | Nanjo | island | 26°10' | 127°45' | 30 | 20 |
| 5 | Kumejima | island | 26°20' | 126°48' | 40 | 20 |
| 6 | Yambaru | island | 26°45' | 128°13' | 37 | 29 |
| 7 | Tokunoshima | island | 27°50' | 128°53' | 50 | 53 |
| 8 | Kikaijima | island | 28°18' | 129°58' | 38 | 20 |
| 9 | Amamioshima | island | 28°27' | 129°36' | 106 | 99 |
| 10 | Takarajima | island | 29°09' | 129°12' | 56 | 57 |
| 11 | Bansyobana | mainland | 31°14' | 130°26' | 20 | 15 |
| 12 | Hioki | mainland | 31°37' | 130°20' | 30 | 15 |
| 13 | Kirishima | mainland | 31°44' | 130°46' | 11 | 10 |
| 14 | Fukuejima | island | 32°41' | 128°45' | 25 | 20 |
| 15 | Nagasaki | mainland | 32°47' | 129°51' | 30 | 24 |
| 16 | Shirahama | mainland | 33°43' | 135°23' | 24 | 36 |
| 17 | Gamouda | mainland | 33°50' | 134°44' | 22 | 23 |
| 18 | Naruto | island | 34°12' | 134°36' | 106 | 102 |
| 19 | Shodoshima | island | 34°29' | 134°11' | 38 | 31 |
| 20 | Tsushima | island | 34°38' | 129°23' | 40 | 22 |
| 21 | Hamada | mainland | 34°57' | 132°08' | 25 | 20 |
| 22 | Imagoura | mainland | 35°39' | 134°38' | 25 | 14 |

Table S2. Correlation between latitude and climate variables.

| Climate variation | Pearson's correlation (*r*) | *P* |
| --- | --- | --- |
|
| Annual mean temperature (°C) | -0.978 | < 0.001 |
| Annual minimum temperature (°C) | -0.977 | < 0.001 |
| Annual maximum temperature (°C) | -0.974 | < 0.001 |
| Annual mean rainfall (mm) | -0.392 | 0.09 |

Table S3. Correlation matrices of five morphological traits. Coefficients for the male and female are shown in the upper and lower triangles, respectively.

|  | EL | FL | FW | TL | TW |
| --- | --- | --- | --- | --- | --- |
| EL |  | 0.869 | 0.588 | 0.952 | 0.869 |
| FL | 0.881 |  | 0.719 | 0.864 | 0.886 |
| FW | 0.772 | 0.888 |  | 0.447 | 0.791 |
| TL | 0.915 | 0.973 | 0.913 |  | 0.795 |
| TW | 0.858 | 0.763 | 0.847 | 0.852 |  |

Table S4. Allometric slopes (± 95％ confidence intervals) of male and female hind leg sizes estimated by SMA regression across 22 populations of *Oedemera sexualis*.

| Number | Population | Trait | Male |  |  | Female |  |  |
| --- | --- | --- | --- | --- | --- | --- | --- | --- |
|  |  |  | *b* | Lower CI | higher CI | *b* | Lower CI | higher CI |
| 1 | Ishigakijima | FL | 1.073 | 0.957 | 1.202 | 1.293 | 1.072 | 1.559 |
|  |  | FW | 1.504 | 1.335 | 1.693 | 1.194 | 0.925 | 1.541 |
|  |  | TL | 1.078 | 0.966 | 1.202 | 1.261 | 1.034 | 1.536 |
|  |  | TW | 2.008 | 1.683 | 2.397 | 1.501 | 1.114 | 2.024 |
| 2 | Iriomotejima | FL | 0.962 | 0.827 | 1.119 | 1.229 | 1.087 | 1.389 |
|  |  | FW | 1.347 | 1.114 | 1.629 | 1.183 | 0.945 | 1.481 |
|  |  | TL | 0.982 | 0.850 | 1.134 | 1.157 | 1.022 | 1.310 |
|  |  | TW | 2.173 | 1.543 | 3.061 | 0.989 | 0.793 | 1.233 |
| 3 | Yonagunijima | FL | 0.911 | 0.855 | 0.971 | 1.121 | 1.058 | 1.189 |
|  |  | FW | 1.395 | 1.294 | 1.505 | 1.210 | 1.103 | 1.328 |
|  |  | TL | 1.008 | 0.942 | 1.080 | 1.144 | 1.045 | 1.252 |
|  |  | TW | 1.872 | 1.694 | 2.068 | 1.371 | 1.241 | 1.516 |
| 4 | Nanjo | FL | 0.936 | 0.842 | 1.039 | 1.122 | 0.971 | 1.297 |
|  |  | FW | 1.477 | 1.271 | 1.716 | 1.249 | 1.021 | 1.528 |
|  |  | TL | 0.983 | 0.874 | 1.106 | 1.016 | 0.879 | 1.173 |
|  |  | TW | 1.912 | 1.584 | 2.306 | 1.344 | 1.138 | 1.587 |
| 5 | Kumejima | FL | 0.877 | 0.797 | 0.964 | 1.161 | 0.969 | 1.391 |
|  |  | FW | 1.289 | 1.164 | 1.428 | 1.246 | 0.932 | 1.666 |
|  |  | TL | 0.949 | 0.865 | 1.041 | 1.173 | 0.983 | 1.399 |
|  |  | TW | 1.366 | 1.168 | 1.596 | 1.588 | 1.213 | 2.078 |
| 6 | Yambaru | FL | 1.080 | 0.943 | 1.238 | 1.162 | 1.061 | 1.272 |
|  |  | FW | 1.407 | 1.226 | 1.616 | 1.262 | 1.100 | 1.448 |
|  |  | TL | 0.969 | 0.869 | 1.081 | 1.200 | 1.074 | 1.340 |
|  |  | TW | 1.611 | 1.325 | 1.959 | 1.199 | 1.000 | 1.436 |
| 7 | Tokunoshima | FL | 0.957 | 0.884 | 1.037 | 1.150 | 1.076 | 1.229 |
|  |  | FW | 1.384 | 1.251 | 1.530 | 1.189 | 1.050 | 1.347 |
|  |  | TL | 0.904 | 0.829 | 0.985 | 1.113 | 1.033 | 1.200 |
|  |  | TW | 1.651 | 1.467 | 1.858 | 1.258 | 1.088 | 1.454 |
| 8 | Kikaijima | FL | 0.883 | 0.795 | 0.980 | 1.079 | 0.892 | 1.305 |
|  |  | FW | 1.288 | 1.114 | 1.490 | 0.984 | 0.718 | 1.350 |
|  |  | TL | 0.956 | 0.834 | 1.095 | 1.007 | 0.833 | 1.217 |
|  |  | TW | 1.553 | 1.343 | 1.796 | 1.230 | 0.843 | 1.794 |
| 9 | Amamioshima | FL | 1.028 | 0.962 | 1.000 | 1.127 | 1.062 | 1.197 |
|  |  | FW | 1.454 | 1.339 | 1.580 | 1.075 | 0.978 | 1.183 |
|  |  | TL | 1.008 | 0.939 | 1.082 | 1.105 | 1.041 | 1.174 |
|  |  | TW | 1.728 | 1.569 | 1.903 | 1.164 | 1.042 | 1.300 |
| 10 | Takarajima | FL | 0.958 | 0.882 | 1.041 | 1.172 | 1.093 | 1.257 |
|  |  | FW | 1.461 | 1.345 | 1.586 | 1.248 | 1.093 | 1.425 |
|  |  | TL | 1.014 | 0.935 | 1.101 | 1.194 | 1.115 | 1.280 |
|  |  | TW | 1.724 | 1.551 | 1.917 | 1.304 | 1.144 | 1.486 |
| 11 | Bansyobana | FL | 0.929 | 0.792 | 1.090 | 1.233 | 1.065 | 1.428 |
|  |  | FW | 1.482 | 1.298 | 1.692 | 1.490 | 1.142 | 1.944 |
|  |  | TL | 1.142 | 0.992 | 1.315 | 1.297 | 1.108 | 1.519 |
|  |  | TW | 1.530 | 1.301 | 1.800 | 1.298 | 0.949 | 1.775 |
| 12 | Hioki | FL | 0.933 | 0.841 | 1.036 | 1.201 | 1.030 | 1.400 |
|  |  | FW | 1.379 | 1.241 | 1.533 | 1.171 | 0.816 | 1.681 |
|  |  | TL | 1.008 | 0.914 | 1.111 | 1.241 | 0.977 | 1.577 |
|  |  | TW | 1.402 | 1.216 | 1.617 | 1.178 | 0.953 | 1.457 |
| 13 | Kirishima | FL | 1.120 | 0.975 | 1.287 | 1.164 | 0.936 | 1.448 |
|  |  | FW | 1.733 | 1.462 | 2.056 | 1.548 | 1.133 | 2.117 |
|  |  | TL | 1.159 | 1.040 | 1.292 | 1.156 | 0.872 | 1.534 |
|  |  | TW | 1.806 | 1.251 | 2.607 | 1.430 | 1.035 | 1.975 |
| 14 | Fukuejima | FL | 1.040 | 0.851 | 1.271 | 1.145 | 0.995 | 1.317 |
|  |  | FW | 1.943 | 1.556 | 2.426 | 1.240 | 0.998 | 1.540 |
|  |  | TL | 1.193 | 0.964 | 1.477 | 1.127 | 0.977 | 1.300 |
|  |  | TW | 2.216 | 1.694 | 2.900 | 1.252 | 1.062 | 1.476 |
| 15 | Nagasaki | FL | 0.904 | 0.800 | 1.021 | 1.091 | 0.998 | 1.192 |
|  |  | FW | 1.397 | 1.216 | 1.604 | 0.980 | 0.800 | 1.199 |
|  |  | TL | 0.972 | 0.831 | 1.136 | 1.046 | 0.958 | 1.142 |
|  |  | TW | 1.568 | 1.284 | 1.914 | 1.119 | 0.950 | 1.318 |
| 16 | Shirahama | FL | 1.129 | 0.963 | 1.323 | 1.254 | 1.156 | 1.359 |
|  |  | FW | 1.600 | 1.323 | 1.934 | 1.383 | 1.128 | 1.694 |
|  |  | TL | 1.086 | 0.965 | 1.222 | 1.212 | 1.107 | 1.340 |
|  |  | TW | 1.685 | 1.415 | 2.006 | 1.335 | 1.115 | 1.598 |
| 17 | Gamouda | FL | 1.131 | 1.000 | 1.278 | 1.278 | 1.154 | 1.415 |
|  |  | FW | 1.824 | 1.610 | 2.067 | 1.250 | 1.052 | 1.484 |
|  |  | TL | 1.283 | 1.126 | 1.461 | 1.207 | 1.076 | 1.354 |
|  |  | TW | 2.032 | 1.672 | 2.469 | 1.391 | 1.129 | 1.715 |
| 18 | Naruto | FL | 0.951 | 0.891 | 1.016 | 1.078 | 1.016 | 1.144 |
|  |  | FW | 1.439 | 1.337 | 1.548 | 1.230 | 1.118 | 1.353 |
|  |  | TL | 1.024 | 0.957 | 1.096 | 1.092 | 1.036 | 1.152 |
|  |  | TW | 1.594 | 1.460 | 1.740 | 1.265 | 1.143 | 1.401 |
| 19 | Shodoshima | FL | 1.080 | 0.925 | 1.261 | 1.062 | 0.941 | 1.200 |
|  |  | FW | 1.498 | 1.299 | 1.729 | 1.417 | 1.211 | 1.658 |
|  |  | TL | 1.007 | 0.829 | 1.224 | 1.029 | 0.892 | 1.188 |
|  |  | TW | 1.808 | 1.503 | 2.176 | 1.407 | 1.147 | 1.726 |
| 20 | Tsushima | FL | 1.014 | 0.905 | 1.136 | 1.083 | 0.978 | 1.199 |
|  |  | FW | 1.728 | 1.558 | 1.916 | 1.219 | 1.046 | 1.421 |
|  |  | TL | 1.073 | 0.955 | 1.206 | 1.095 | 0.979 | 1.224 |
|  |  | TW | 1.804 | 1.575 | 2.067 | 1.329 | 1.066 | 1.658 |
| 21 | Hamada | FL | 0.934 | 0.805 | 1.085 | 0.925 | 0.823 | 1.041 |
|  |  | FW | 1.440 | 1.233 | 1.681 | 1.237 | 0.952 | 1.607 |
|  |  | TL | 0.973 | 0.836 | 1.132 | 0.899 | 0.780 | 1.036 |
|  |  | TW | 1.605 | 1.309 | 1.968 | 1.366 | 1.010 | 1.848 |
| 22 | Imagoura | FL | 1.020 | 0.917 | 1.135 | 1.121 | 0.959 | 1.310 |
|  |  | FW | 1.428 | 1.325 | 1.539 | 1.189 | 0.953 | 1.484 |
|  |  | TL | 1.087 | 0.971 | 1.217 | 1.106 | 0.952 | 1.284 |
|  |  | TW | 1.514 | 1.299 | 1.765 | 1.161 | 0.953 | 1.414 |
